# Supplementary material for: Eco-evolutionary feedbacks in the human gut microbiome
Source: Nat Commun. 2023 Nov 6;14:7146. doi: 10.1038/s41467-023-42769-3 (PMC10628149; doi:10.1038/s41467-023-42769-3)
Supplement: Supplementary file 1 — Supplementary Information [file 41467_2023_42769_MOESM1_ESM.pdf]

Supplementary Notes

Supplementary Note 1: Eco-evolutionary feedbacks in saturated resource competition models

To develop intuition for the potential mechanisms that could produce the data in Figs. 1-3, we studied the eco-evolutionary correlations that arise in a simple mathematical model of an evolving microbial community. We focused on a simple class of resource competition models (46) of the form studied in Ref. (15) and other works (45, 48, 49). We assume that microbial cells compete within a well-mixed, chemostat-like environment, in which $\mathcal{R}$ substitutable resources are supplied at constant flux, with β_i denoting the fraction of biomass supplied in the form of resource i. We assume that species take up these resources at different genetically encoded rates, $r_{\mu,i}$. We previously showed (15) that it can be useful to decompose these uptake rates into a normalized resource strategy,

$$\alpha_{\mu,i} = \frac{r_{\mu,i}}{\sum_j r_{\mu,j}}\,, \tag{S1.1}$$

which represents the fraction of effort devoted to acquiring resources of type i, as well as a total uptake rate,

$$X_\mu = \log\left(\sum_j r_{\mu,i}\right)\,, \tag{S1.2}$$

which we will call the *general fitness* component. In this notation, the resulting ecological dynamics can then be written in a compact form,

$$\frac{\partial f_\mu}{\partial t} = \left[-1 + \sum_{i=1}^{\mathcal{R}} \frac{\beta_i \alpha_{\mu,i} e^{X_\mu}}{\sum_\nu \alpha_{\nu,i} e^{X_\nu} f_\nu}\right] f_\mu + \text{noise}, \tag{S1.3}$$

where f_μ denotes the relative abundance of species μ (15). At long times, these dynamics approach a unique stable fixed point (*ecological equilibrium*) with at most $\mathcal{R}$ coexisting species.

Here we focus on a scenario in which the community is already at its ecological equilibrium, and a new mutation occurs in one of the $\mathcal{S}$ resident species. In principle, this initial collection of species could be produced by a complex assembly process, involving a mixture of habitat filtering, host selection, and/or previous local evolution. Here, we will focus on the special case of a *saturated community* ($\mathcal{S} = \mathcal{R}$), where the ecological equilibria are particularly well characterized. We previously showed that the relative abundances in such an ecosystem are given by

$$f_\mu = \sum_{i=1}^{\mathcal{R}} \frac{\beta_i \alpha^{-1}_{i,\mu}}{1 + \sum_\nu \alpha^{-1}_{i,\nu}\left(e^{X_\mu - X_\nu} - 1\right)}\,, \tag{S1.4}$$

where $\alpha^{-1}_{i,\mu}$ is the inverse of $\alpha_{\mu,i}$. The normalization of the resource strategies ($\sum_i \alpha_{\mu,i} = 1$) implies that $\sum_\mu \alpha^{-1}_{i,\mu} = 1$. Much of the relevant behavior can be observed for a special class of resource strategies,

$$\alpha_{\mu,i} = \beta_i(1-\epsilon) + \epsilon \cdot \tilde{\alpha}_{\mu,i}\,, \tag{S1.5}$$

where $\tilde{\alpha}_{\mu,i}$ is another normalized resource strategy and ϵ is a small parameter. In this case, the inverse of $\alpha_{\mu,i}$ is asymptotically given by

$$\alpha^{-1}_{i,\mu} = \frac{\tilde{\alpha}^{-1}_{i,\mu} - \sum_i \beta_i \tilde{\alpha}^{-1}_{i,\mu}}{\epsilon} + \sum_i \beta_i \tilde{\alpha}^{-1}_{i,\mu} + \mathcal{O}(\epsilon)\,. \tag{S1.6}$$

If we define a corresponding rescaling of the overall fitness, $X_\mu = \epsilon^2 \tilde{X}_\mu$, then we can expand Eq. (S1.4) in the limit of small ϵ to obtain

$$f_\mu = \sum_i \beta_i \tilde{\alpha}^{-1}_{i,\mu} + \sum_{i,j,k,\nu} \beta_i \beta_j \beta_k \left(\tilde{\alpha}^{-1}_{i,\mu} \tilde{\alpha}^{-1}_{i,\nu} - \tilde{\alpha}^{-1}_{j,\mu} \tilde{\alpha}^{-1}_{k,\nu} \right) \tilde{X}_\nu + \mathcal{O}(\epsilon) \, , \tag{S1.7}$$

This expression can also be written in the compact form,

$$f_\mu = \mathbb{E}_\beta[\alpha^{-1}_\mu] + \sum_\nu \mathrm{Cov}_\beta(\alpha^{-1}_\mu, \alpha^{-1}_\nu) X_\nu + \mathcal{O}(\epsilon) \, , \tag{S1.8}$$

where $\mathbb{E}_\beta[\alpha^{-1}_\mu]$ and $\mathrm{Cov}_\beta(\alpha^{-1}_\mu, \alpha^{-1}_\nu)$ denote particular moments of the inverse matrix:

$$\mathbb{E}_\beta[\alpha^{-1}_\mu] \equiv \sum_i \beta_i \alpha^{-1}_{i,\mu} \, , \tag{S1.9}$$

$$\mathrm{Cov}_\beta(\alpha^{-1}_\mu, \alpha^{-1}_\nu) \equiv \sum_i \beta_i \alpha^{-1}_{i,\mu} \alpha^{-1}_{i,\nu} - \mathbb{E}_\beta[\alpha^{-1}_\mu] \cdot \mathbb{E}_\beta[\alpha^{-1}_\nu] \, , \tag{S1.10}$$

which are weighted by the external resource supply rates β_i.

Within such a community, we wish to examine the invasion fitness and ecological impact of a mutation that changes the phenotype of a focal species (μ^*). These mutations can change the resource strategy $\alpha_{\mu,i}$, the overall fitness component X_μ, or a combination of the two. We can write this as:

$$X_{\mu^*} \to X_{\mu^*} + \Delta X \, , \quad \alpha_{\mu^*,i} \to \alpha_{\mu^*,i} + \gamma_i \, , \tag{S1.11}$$

where γ_i is normalized so that $\sum_i \gamma_i = 0$. We previously showed (15) that the invasion fitness of such a mutation is given by

$$S_{\mathrm{inv}} = \Delta X - \sum_{i=1}^{\mathcal{R}} \gamma_i \overline{X}_i \, , \tag{S1.12}$$

where $\overline{X}_i = \sum_\mu \alpha^{-1}_{i,\mu} X_\mu$. By defining scaled versions of these quantities ($S_{\mathrm{inv}} = \epsilon^2 \tilde{S}_{\mathrm{inv}}$, $\Delta X = \epsilon^2 \Delta \tilde{X}$, and $\gamma_i = \epsilon \tilde{\gamma}_i$), this reduces to

$$\tilde{S}_{\mathrm{inv}} \approx \Delta \tilde{X} - \sum_{i,\mu} \tilde{\gamma}_i \tilde{\alpha}^{-1}_{i,\mu} \tilde{X}_\mu + \mathcal{O}(\epsilon) \tag{S1.13}$$

in the limit that $\epsilon \ll 1$ (15). Note that this expression is independent of the external resource supply rates β_i. This shows that, in saturated communities at ecological equilibrium, the selection pressures on new mutations are independent of the external environment. This occurs because saturated communities can dynamically readjust their species composition in Eq. (S1.4) in such a way that the internal resource concentrations remain constant (15, 49). In this way, the collective behavior of the community will tend to shield it from any ecology-driven feedbacks, provided that the timescales of the external fluctuations are long compared to the internal equilibration time. This provides a proof-of-principle example of a large community in which only evolutionary feedbacks are possible. We will consider these feedbacks for different classes of mutations below.

Pure fitness mutations. We first consider the case where the mutation increases the overall fitness of the strain ($\Delta X > 0$) but leaves its resource strategy intact ($\gamma_i = 0$). The invasion fitness of such a mutation is simply given by

$$\tilde{S}_{\mathrm{inv}} = \Delta \tilde{X} \quad \implies \quad S_{\mathrm{inv}} = \Delta X \, . \tag{S1.14}$$

If this mutation establishes, it will outcompete its ancestor and alter the ecological equilibrium between species. For sufficiently small $\Delta\tilde{X}$, none of the other species will go extinct, and the new equilibrium can be calculated from Eq. (S1.4) by perturbing $\tilde{X}_\mu \to \tilde{X}_\mu + \Delta\tilde{X}\delta_{\mu,\mu^*}$. The corresponding relative abundance changes are given by

$$\Delta f_\mu = \Delta\tilde{X} \times \sum_{i,j,k} \beta_i\beta_j\beta_k \left(\tilde{\alpha}^{-1}_{i,\mu}\tilde{\alpha}^{-1}_{i,\mu^*} - \tilde{\alpha}^{-1}_{j,\mu}\tilde{\alpha}^{-1}_{k,\mu^*}\right) , \tag{S1.15}$$

which can also be written as

$$\Delta f_\mu = S_{\text{inv}} \left(\frac{\text{Cov}_\beta(\tilde{\alpha}^{-1}_\mu, \tilde{\alpha}^{-1}_{\mu^*})}{\epsilon^2}\right) , \tag{S1.16}$$

where $\text{Cov}_\beta(\tilde{\alpha}^{-1}_\mu, \tilde{\alpha}^{-1}_{\mu^*})$ is defined as in Eq. (S1.10) above. This shows that for a given invasion fitness S_{inv} (e.g. as measured in metagenomic sequencing), larger shifts in frequency occur in communities with a larger degree of metabolic overlap (smaller ϵ). Conversely, for a fixed set of resource strategies, larger invasion fitnesses result in larger ecological perturbations. Furthermore, since

$$\text{Cov}_\beta(\tilde{\alpha}^{-1}_{\mu^*}, \tilde{\alpha}^{-1}_{\mu^*}) = \text{Var}_\beta(\tilde{\alpha}^{-1}_{\mu^*}) \geq 0 \, , \tag{S1.17}$$

Eq. (S1.16) shows that the shift in the focal strain is always positive for a pure fitness mutation. The ratio of the shifts in frequency can be written as

$$\frac{\Delta f_\mu}{\Delta f_{\mu^*}} = \frac{\text{Cov}_\beta(\tilde{\alpha}^{-1}_\mu, \tilde{\alpha}^{-1}_{\mu^*})}{\text{Var}_\beta(\tilde{\alpha}^{-1}_{\mu^*})} \, , \tag{S1.18}$$

which is independent of S_{inv}, ϵ, and X_μ, and depends only on the strategy covariance matrices defined above. The right hand side of Eq. (S1.18) is reminiscent of some classical measures of niche overlap computed from the correlations of resource utilization vectors (47). In this case, however, because the covariances depend on the inverse matrix ($\alpha^{-1}_{i,\mu}$) rather than the resource strategies themselves, the abundance fluctuations will generally depend on the resource strategies of the entire community (as opposed to μ and μ^* alone).

Pure strategy mutations. We next consider the case where the mutation alters the resource strategy of the strain ($\gamma_i \neq 0$) but leaves its overall fitness component intact ($\Delta X = 0$). Equation (S1.13) shows that the invasion fitness of such a mutation is given by

$$\tilde{S}_{\text{inv}} \approx -\sum_{i,\mu} \tilde{\gamma}_i \tilde{\alpha}^{-1}_{i,\mu}\tilde{X}_\mu \, . \tag{S1.19}$$

This shows that only a subset of these mutations will be favored by selection ($S_{\text{inv}} > 0$). As above, we wish to understand how a successful strategy mutation changes the ecological equilibrium at steady state. In the limit of small $\tilde{\gamma}_i$, the mutation will outcompete its parent strain and lead to small changes in the frequencies of the other strains. This results in a new set of resource strategy vectors,

$$\tilde{\alpha}'_{\mu,i} = \tilde{\alpha}_{\mu,i} + \gamma_i \delta_{\mu,\mu^*} \, . \tag{S1.20}$$

We can calculate the new equilibrium frequencies from Eq. (S1.4) with the help of the identity,

$$(\tilde{\alpha}'_{i,\mu})^{-1} \approx \tilde{\alpha}^{-1}_{i,\mu} - \sum_{j,\nu} \tilde{\alpha}^{-1}_{i,\nu}\tilde{\gamma}_j\delta_{\nu,\mu^*}\tilde{\alpha}^{-1}_{j,\mu} \approx \tilde{\alpha}^{-1}_{i,\mu} - \tilde{\alpha}^{-1}_{i,\mu^*}\sum_j \tilde{\gamma}_j \tilde{\alpha}^{-1}_{j,\mu} \, . \tag{S1.21}$$

Substituting into Eq. (S1.4) , we obtain

$$\begin{aligned}\Delta f_\mu = -f^0_{\mu^*}\sum_j \tilde{\gamma}_j\tilde{\alpha}^{-1}_{j,\mu} - \sum_{i,j,\nu}\beta_i\tilde{\alpha}^{-1}_{i,\mu}\tilde{\alpha}^{-1}_{i,\mu^*}\cdot\tilde{\gamma}_j\tilde{\alpha}^{-1}_{j,\nu}\tilde{X}_\nu - \sum_{i,j,\nu}\beta_i\tilde{\alpha}^{-1}_{i,\nu}\tilde{\alpha}^{-1}_{i,\mu^*}\cdot\tilde{\gamma}_j\tilde{\alpha}^{-1}_{j,\mu}\tilde{X}_\nu \\ + \sum_{\nu,j} f^0_{\mu^*}f^0_\nu\tilde{\gamma}_j\tilde{\alpha}^{-1}_{j,\mu}\tilde{X}_\nu + \sum_{\nu,j} f^0_{\mu^*}f^0_\mu\tilde{\gamma}_j\tilde{\alpha}^{-1}_{j,\nu}\tilde{X}_\nu + \mathcal{O}(\tilde{\gamma}^2)\,,\end{aligned} \tag{S1.22}$$

where $f^0_\mu = \mathbb{E}_\beta[\alpha^{-1}_\mu]$ is the equilibrium frequency in the absence of any fitness differences ($X_\mu = 0$). Using the definition of the invasion fitness in Eq. (S1.19), we can write expression in the compact form,

$$\Delta f_\mu = -f_{\mu^*}\sum_j \tilde{\gamma}_j\tilde{\alpha}^{-1}_{j,\mu} + \tilde{S}_{\text{inv}}\left[\sum_{i,j}\beta_i\beta_j\left(\tilde{\alpha}^{-1}_{i,\mu}\tilde{\alpha}^{-1}_{i,\mu^*} - \tilde{\alpha}^{-1}_{i,\mu}\tilde{\alpha}^{-1}_{j,\mu^*}\right)\right]\,. \tag{S1.23}$$

We can then turn this expression around to solve for $\tilde{\gamma}_i$ as a function of Δf_μ:

$$\tilde{\gamma}_i = -\frac{1}{f_{\mu^*}}\sum_\mu \Delta f_\mu\tilde{\alpha}_{\mu,i} + \frac{\tilde{S}_{\text{inv}}}{f_{\mu^*}}\sum_\mu \tilde{\alpha}_{\mu,i}\left[\sum_{i,j}\beta_i\beta_j\left(\tilde{\alpha}^{-1}_{i,\mu}\tilde{\alpha}^{-1}_{i,\mu^*} - \tilde{\alpha}^{-1}_{i,\mu}\tilde{\alpha}^{-1}_{j,\mu^*}\right)\right]\,. \tag{S1.24}$$

Plugging back in to our expression for $\tilde{S}_{\text{inv}}$ in Eq. (S1.19), we have a self-consistent solution for $\tilde{S}_{\text{inv}}$:

$$\begin{aligned}\tilde{S}_{\text{inv}} &= \frac{1}{f_{\mu^*}}\sum_{i,\nu,\mu}\tilde{\alpha}^{-1}_{i,\nu}X_\nu\Delta f_\mu\tilde{\alpha}_{\mu,i} - \frac{\tilde{S}_{\text{inv}}}{f_{\mu^*}}\sum_{k,\nu,\mu}\tilde{\alpha}^{-1}_{k,\nu}X_\nu\tilde{\alpha}_{\mu,k}\left[\sum_{i,j}\beta_i\beta_j\left(\tilde{\alpha}^{-1}_{i,\mu}\tilde{\alpha}^{-1}_{i,\mu^*} - \tilde{\alpha}^{-1}_{i,\mu}\tilde{\alpha}^{-1}_{j,\mu^*}\right)\right]\,, \\ &= \frac{1}{f_{\mu^*}}\sum_\mu \Delta f_\mu\tilde{X}_\mu - \frac{\tilde{S}_{\text{inv}}}{f_{\mu^*}}\sum_\mu X_\mu\left[\sum_{i,j}\beta_i\beta_j\left(\tilde{\alpha}^{-1}_{i,\mu}\tilde{\alpha}^{-1}_{i,\mu^*} - \tilde{\alpha}^{-1}_{i,\mu}\tilde{\alpha}^{-1}_{j,\mu^*}\right)\right]\,,\end{aligned} \tag{S1.25}$$

where we have used the fact that $\sum_i \tilde{\alpha}^{-1}_{i,\nu}\tilde{\alpha}_{\mu,i} = \delta_{\mu,\nu}$. Solving for $\tilde{S}_{\text{inv}}$ and converting back to unscaled fitnesses, we find that

$$S_{\text{inv}} = \frac{\sum_\mu \Delta f_\mu\cdot X_\mu}{2f_{\mu^*} - f^0_{\mu^*}}\,. \tag{S1.26}$$

Note that the sign of this expression depends on the focal species only through the factor $2f_{\mu^*} - f^0_{\mu^*}$, which is independent of the new mutation. When $2f_{\mu^*} - f^0_{\mu^*} > 0$, natural selection will favor mutations that increase the community-level quantity $\sum_\mu f_\mu\cdot X_\mu$, even if the focal species itself declines in abundance. Conversely, if $2f_{\mu^*} - f^0_{\mu^*} < 0$, natural selection will favor mutations that lead to lower values of $\sum f_\mu\cdot X_\mu$, independent of the focal species change.

General mutations. We can combine the results above to understand what happens for a general mutation. At linear order, we still obtain

$$\Delta f_\mu = -f_{\mu^*}\sum_j \tilde{\gamma}_j\tilde{\alpha}^{-1}_{j,\mu} + \tilde{S}_{\text{inv}}\left[\sum_{i,j}\beta_i\beta_j\left(\tilde{\alpha}^{-1}_{i,\mu}\tilde{\alpha}^{-1}_{i,\mu^*} - \tilde{\alpha}^{-1}_{i,\mu}\tilde{\alpha}^{-1}_{j,\mu^*}\right)\right] \tag{S1.27}$$

where $\tilde{S}_{\rm inv}$ now includes the contribution from $\Delta\tilde{X}$. Solving for $\tilde{\gamma}_i$ and plugging into Eq. (S1.19), we obtain

$$\tilde{S}_{\rm inv} = \Delta\tilde{X} + \frac{1}{f_{\mu^*}}\sum_\mu \Delta f_\mu \tilde{X}_\mu - \frac{\tilde{S}_{\rm inv}}{f_{\mu^*}}\sum_\mu X_\mu \left[\sum_{i,j}\beta_i\beta_j\left(\tilde{\alpha}^{-1}_{i,\mu}\tilde{\alpha}^{-1}_{i,\mu^*} - \tilde{\alpha}^{-1}_{i,\mu}\tilde{\alpha}^{-1}_{j,1}\right)\right], \tag{S1.28}$$

which yields the general expression,

$$S_{\rm inv} = \frac{\Delta X f_{\mu^*} + \sum_\mu \Delta f_\mu X_\mu}{2f_{\mu^*} - f^0_{\mu^*}}, \tag{S1.29}$$

quoted in Eq. (1) in the main text.

Example for two species. We can gain some further intuition for this result by focusing on the case where $\mathcal{S} = \mathcal{R} = 2$, which is illustrated in Fig. 4C. Without loss of generality, we can label the two species so that the focal species is $\mu^* = 1$. In this case, the inverse resource strategy matrix can be computed exactly:

$$\alpha^{-1}_{i,\mu} = \frac{1}{\Delta\alpha}\begin{pmatrix} 1-\alpha_2 & -(1-\alpha_1) \\ -\alpha_2 & \alpha_1 \end{pmatrix}, \tag{S1.30}$$

so that

$$S_{\rm inv} = s - \frac{\gamma\Delta X}{\Delta\alpha}, \tag{S1.31}$$

$$f^0_1 = \sum_i \beta_i \alpha^{-1}_{i,1} = \frac{\beta - \alpha_2}{\Delta\alpha}, \tag{S1.32}$$

and

$$f_1 = \frac{\beta - \alpha_2}{\Delta\alpha} + \frac{\beta(1-\beta)\Delta X}{\Delta\alpha^2}. \tag{S1.33}$$

The change in frequency from an infinitesimal γ is given by

$$\Delta f \approx f^0_1 \cdot \frac{-\gamma}{\Delta\alpha} + \frac{\beta(1-\beta)\Delta X}{\Delta\alpha^2}\frac{-2\gamma}{\Delta\alpha}, \tag{S1.34}$$

or

$$\Delta f = -\left(2f_1 - f^0_1\right)\frac{\gamma}{\Delta\alpha}. \tag{S1.35}$$

These results show that for a fixed γ, the fitness landscape is independent of the sign of $2f_1 - f^0_1$. If $\Delta X > 0$, then there is always selection pressure for $\gamma > 0$. In this case, $f_1 > f^0_1$, so the corresponding change Δf is always positive. Conversely, if $\Delta X < 0$, then there is always selection pressure for $\gamma < 0$. In this case, the sign of Δf depends on f_1 vs f^0_1. If $f_1 > f^0_1/2$, then the mutation leads to $\Delta f < 0$, and an increase in $\sum_\mu f_\mu X_\mu$. However, if $f_1 < f^0_1/2$, then the mutation leads to $\Delta f > 0$, and a decrease in the community-wide $\sum_\mu f_\mu X_\mu$.

Supplementary Figures

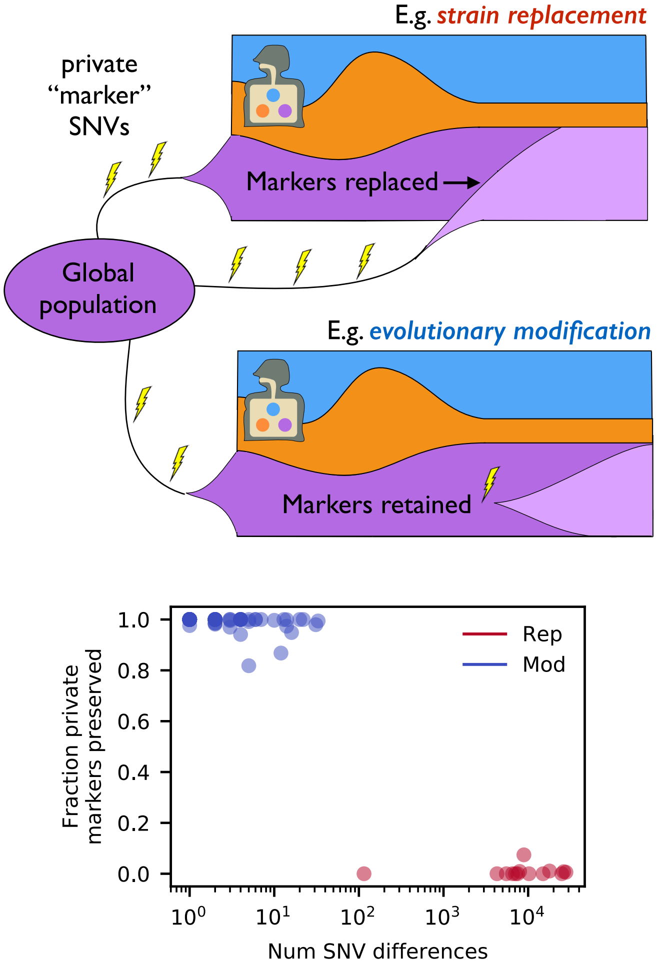

Supplementary Figure 1: Schematic illustration of strain replacement and evolutionary modification events within a host. In the former case, private marker SNVs that are fixed in the resident population at the initial timepoint are replaced when an external strain sweeps through the population (4, 9). In contrast, private marker SNVs are preserved in an evolutionary modification event, when mutations accumulate on the background of the resident strain. Bottom panel shows the fraction of private marker SNVs preserved as a function of the total number of SNV differences for all of the populations in our study with at least 10 private marker SNVs. The sharp transition at ≈100 SNVs motivates our operational definition of a strain replacement event as one with >100 SNV differences between timepoints (Methods).

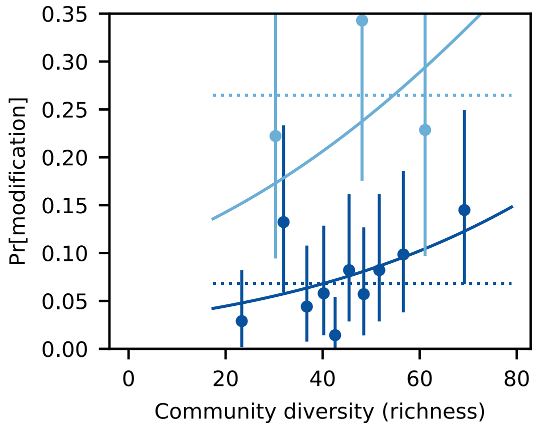

Supplementary Figure 2: An analogous version of Fig. 1C using species richness as a measure of community diversity. Richness was calculated as $\mathcal{S} = \sum_\mu [1 - \exp(-f_\mu/f_0)]$ with $f_0 = 10^{-3}$. This metric approximates the number of species with relative abundance $\gtrsim 10^{-3}$, but is less sensitive to the presence of log-scale fluctuations (33) near the minimum frequency threshold.

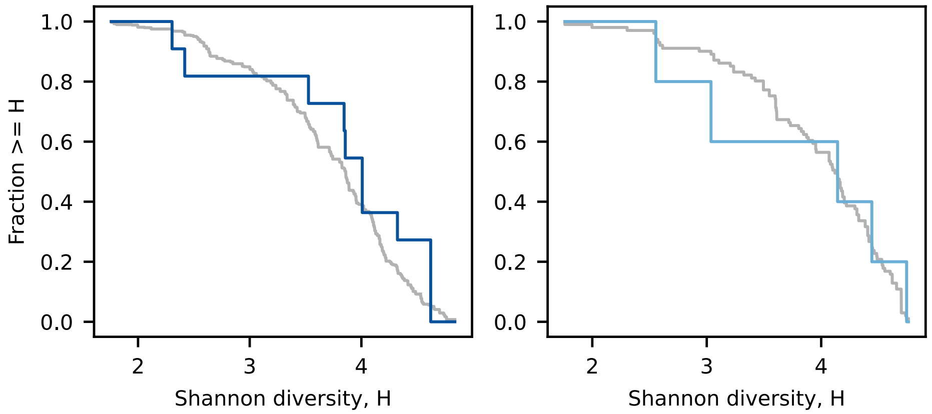

Supplementary Figure 3: Distribution of Shannon entropy at the initial timepoint for all species that experienced a strain replacement event. Bacteroidetes species are shown on the left, while Firmicutes species are shown on the right. Colored lines denote the observed data, while grey lines denote the background distribution for all resident populations from the corresponding phylum. These data show that strain replacements are not enriched for higher levels of initial community diversity, consistent with the logistic regression results in Supplementary Table 1.

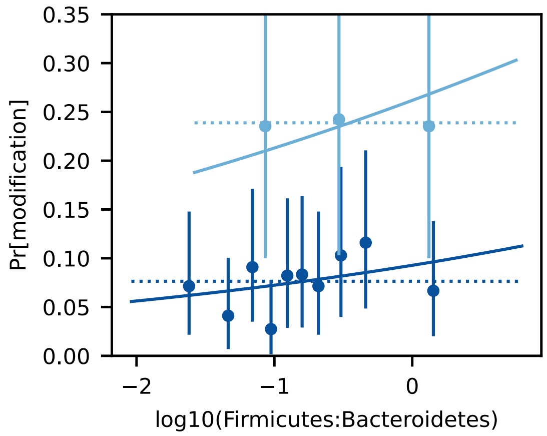

Supplementary Figure 4: An analogous version of Fig. 1C as a function of the Firmicutes-to-Bacteroidetes ratio. The F/B ratio was computed by summing the relative abundances of the species in the Firmicutes and Bacteroidetes phyla at the initial timepoint. We observed no systematic correlation between the F/B ratio and the rate of evolutionary modification, after controlling for the phylum of the focal species ($P \approx 0.3$; logistic regression). Similar results were observed for the rate of strain replacement ($P \approx 0.2$). These results suggests that the F/B ratio does not significantly impact the rate of within-host evolution in our dataset.

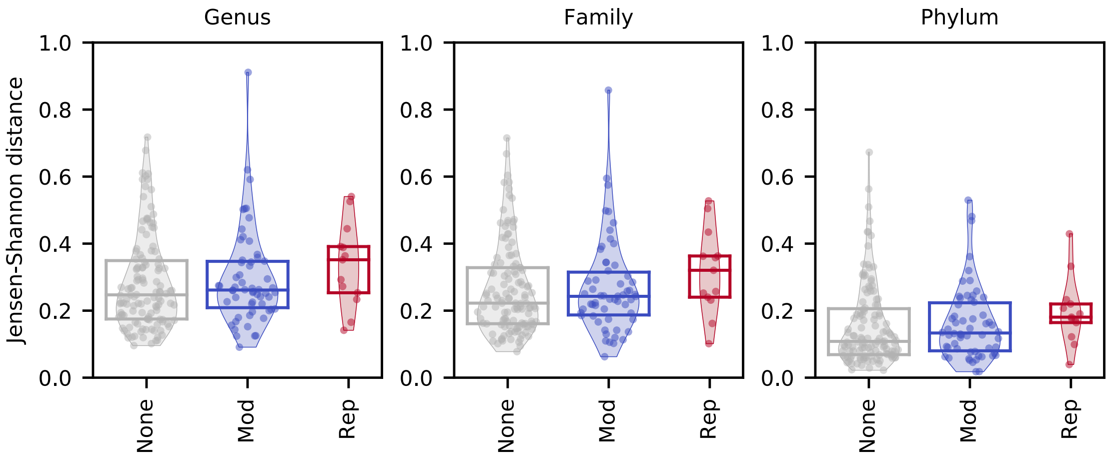

Supplementary Figure 5: Analogous versions of Fig. 2D using Jensen-Shannon distances computed from species abundances coarse-grained at the genus, family, and phylum levels. The corresponding P-values of the one-sided KS tests (Methods) are $P_{\mathrm{mod}} \approx 0.01$, $P_{\mathrm{rep}} \approx 0.01$ (genus-level), $P_{\mathrm{mod}} \approx 0.02$, $P_{\mathrm{rep}} \approx 0.001$ (family-level), $P_{\mathrm{mod}} \approx 0.01$, $P_{\mathrm{rep}} \approx 0.001$ (phylum-level). Sample sizes for each category are the same as in Fig. 2D.

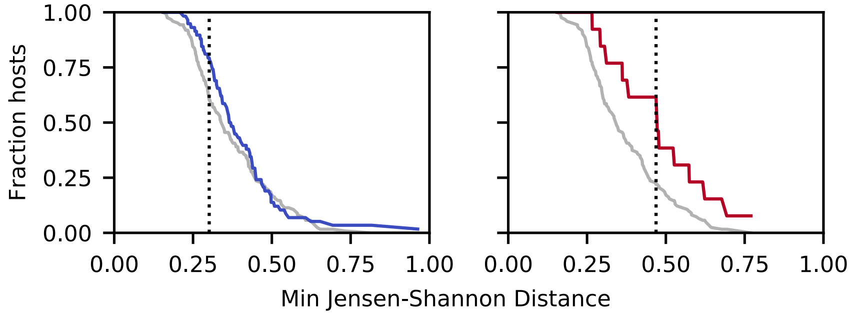

Supplementary Figure 6: Survival distributions for the Jensen-Shannon distances in Fig. 2D, illustrating the one-sided Kolmogorov-Smirnov (KS) test (Methods). The dashed line shows the location of the maximum deviation between the two deviations, which defines the KS statistic D in Eq. (7).

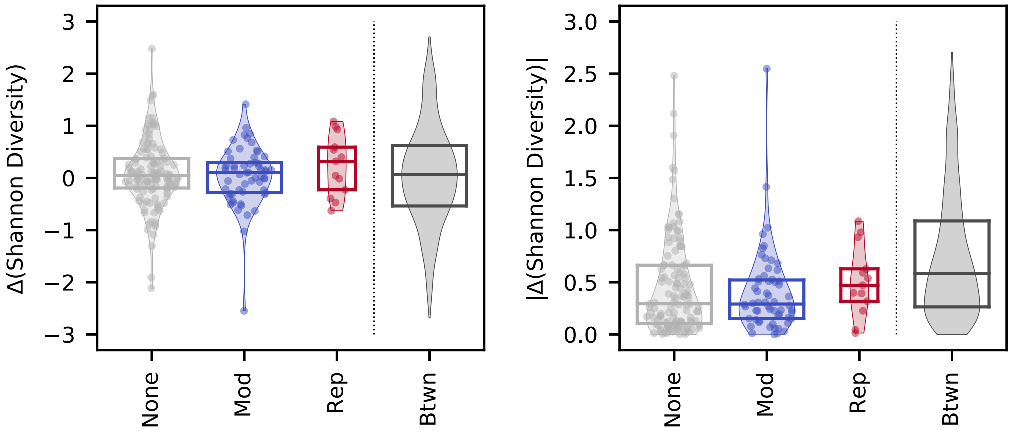

Supplementary Figure 7: An analogous version of Fig. 2D using the change in Shannon diversity (left) or its absolute value (right) as an ecological distance metric. The only (marginally) significant difference occurs for the absolute value of the replacement events ($P \approx 0.05$, Methods); all other comparisons have $P > 0.1$. Sample sizes for each category are the same as in Fig. 2D.

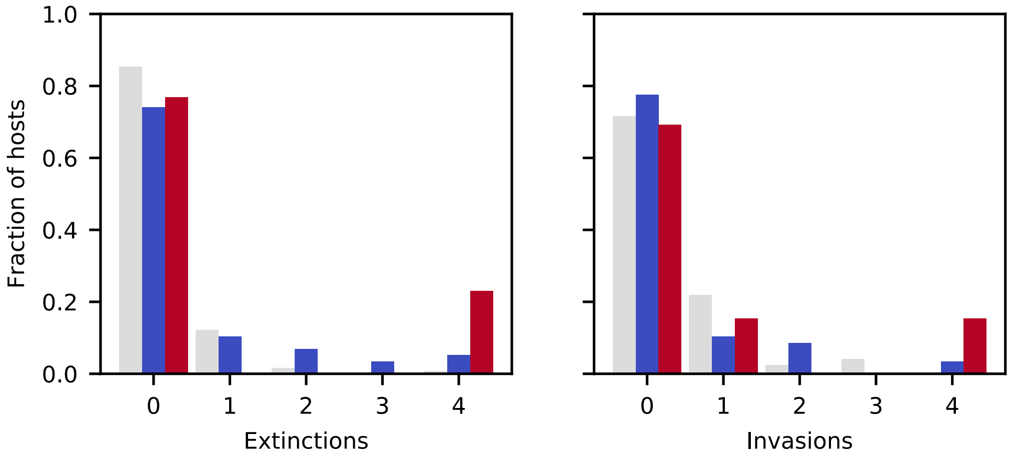

Supplementary Figure 8: An analogous version of Fig. 2G showing "invasion" as well as "extinction" events of abundant species (Methods). Invasions are defined as the time-reversed version of an extinction event, so that the relative numbers of extinction and invasion events can be compared. In contrast to extinction events, replacement and modifications do not show a significantly increased number of invasions relative to hosts with no genetic changes ($P_{\rm rep} \approx 0.08$ and $P_{\rm mod} \approx 0.2$, Methods). Interestingly, however, hosts with no genetic changes have a significantly increased fraction of invasions vs extinctions ($P \approx 0.01$, Methods), indicating a violation of time-reversal symmetry.

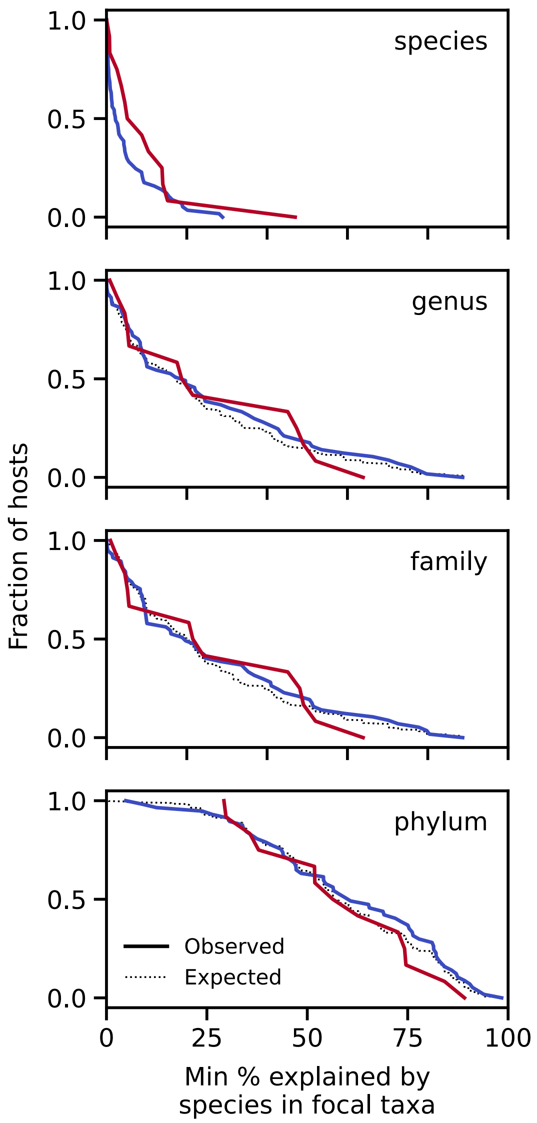

Supplementary Figure 9: Analogous versions of Fig. 3E,F computed for a range of taxonomic distances from the focal species. We observed a small enrichment in the average JSD explained by species in the same genus for evolutionary modification events (uncorrected $P \approx 0.02$, effect size=0.04, Methods). All other comparisons were not statistically significant.

Supplementary Tables

Regression model	n	n_1	β_{phylum}	P_{phylum}	β_{H_0}	P_{H_0}
$\text{logit}\, p(\text{rep}) \sim \text{phylum} + H_0$	784	16	1.136	0.041	0.095	0.807
$\text{logit}\, p(\text{mod}) \sim \text{phylum} + H_0$	784	76	1.247	<0.001	0.421	0.037
$\text{logit}\, p(\text{mod}) \sim \text{phylum} + H_0$ (bottom 90% of H_0)	705	62	1.247	<0.001	0.421	0.037
$\text{logit}\, p(\text{rep}) \sim \text{phylum} + S_0$	784	16	1.102	0.047	0.018	0.425
$\text{logit}\, p(\text{mod}) \sim \text{phylum} + S_0$	784	76	1.270	<0.001	0.022	0.044

Supplementary Table 1: Results of logistic regressions comparing the rates of strain replacement and evolutionary modification in different species populations as a function of the initial community diversity (Methods), as quantified by Shannon diversity (H_0) or the richness metric in Supplementary Fig. 2 (S_0). From left to right, columns list the regression model, the total sample size (n), the total number of populations in which the event was observed (n_1), the regression coefficients (β) and two-sided P-values for the phylum of the focal species (either Bacteroidetes or Firmicutes) and the community diversity metric, respectively. No adjustments were made for multiple comparisons.
