# Supplementary material for: Eco-evolutionary feedbacks in the human gut microbiome
Source: Nat Commun. 2023 Nov 6;14:7146. doi: 10.1038/s41467-023-42769-3 (PMC10628149; doi:10.1038/s41467-023-42769-3)
Supplement: Supplementary file 3 — Description of Additional Supplementary Files [file 41467_2023_42769_MOESM3_ESM.pdf]

Supplementary Data Files

Supplementary Data 1. List of metagenomic samples and metadata used in this study.

Supplementary Data 2. Results of sweep-detection pipeline applied to all non-redundant pairs of quasi-phaseable timepoints.

Supplementary Data 3. Locations of SNVs for all detected sweeps in Supplementary Data 2.

Supplementary Data 4. Species relative abundance estimates for each of the samples in Supplementary Data 1.

Supplementary Code 1. Analysis code and figure generation scripts for Figures 1-3.
